# Salt-Related Knowledge, Attitudes and Behavior in an Intervention to Reduce Added Salt When Cooking in a Sample of Adults in Portugal

**DOI:** 10.3390/foods11070981

**Published:** 2022-03-28

**Authors:** Tânia Silva-Santos, Pedro Moreira, Olívia Pinho, Patrícia Padrão, Pedro Norton, Carla Gonçalves

**Affiliations:** 1 Faculty of Nutrition and Food Sciences, University of Porto, 4150-180 Porto, Portugal; pedromoreira@fcna.up.pt (P.M.); oliviapinho@fcna.up.pt (O.P.); patriciapadrao@fcna.up.pt (P.P.); carlagoncalves.pt@gmail.com (C.G.); 2 EPI Unit, Institute of Public Health, University of Porto, 4200-450 Porto, Portugal; pnorton@med.up.pt; 3 ITR—Laboratory for Integrative and Translational Research in Population Health, Institute of Public Health, 4050-600 Porto, Portugal; 4 LAQV-REQUIMTE—Laboratory of Bromatology and Hydrology, Faculty of Pharmacy, University of Porto, 5000-801 Porto, Portugal; 5 Faculty of Medicine, University of Porto, 4200-319 Porto, Portugal; 6 Occupational Health Service, Centro Hospitalar Universitário de São João, 4200-319 Porto, Portugal; 7 CITAB—Centre for the Research and Technology of Agro-Environmental and Biological Sciences, University of Trás-os-Montes and Alto Douro, 5000-801 Vila Real, Portugal

**Keywords:** dietary salt, dietary sodium, cardiovascular disease, salt-related knowledge, KAB

## Abstract

(1) Background: Excessive salt intake is associated with an increased risk of hypertension and cardiovascular disease, so reducing it is critical. The main objective of this study was to verify whether one intervention to reduce added salt during cooking changed knowledge, attitudes and behavior (KAB) towards salt, and to analyze changes in the main sources of salt. (2) Methods: The intervention study was an 8-week randomized controlled trial with 97 workers from a public university. KAB in relation to salt were obtained through the WHO STEPwise questionnaire, and the main sources of salt were obtained by 24-h food recall and 24 h urinary sodium excretion over two days. (3) Results: After the intervention, participants in the intervention group reported a decrease in the addition of salt when cooking (*p* = 0.037), an increase in the percentage of subjects who avoided the consumption of processed foods (from 54.2% to 83.3%, *p* = 0.001), who looked for salt on food labels (from 18.8% to 39.6%, *p* = 0.013), and who bought low-salt food alternatives (from 43.8% to 60.4%, *p* = 0.039). However, there were no significant differences between the intervention group and the control group at baseline and post-intervention assessments. In the intervention group, after the intervention, the added salt decreased by 5%; food sources of salt such as the snacks and pizza group decreased by 7%, and the meat, fish and eggs group increased by 4%, but without statistical significance. (4) Conclusions: With innovative equipment for dosing salt when cooking, it is possible to change some dimensions of consumer behavior in relation to salt.

## 1. Introduction

About 17 million people die every year from cardiovascular disease and approximately 9.4 million are from complications of hypertension [1,2].

Strong evidence has shown a causal relationship between sodium intake and blood pressure [3,4,5,6,7,8]. A modest reduction in sodium causes a reduction in blood pressure in hypertensive and normotensive individuals with clinical and public health significance [9,10]. Sodium is an essential nutrient, and the most common form of sodium consumption is sodium chloride, consisting of 40% sodium, and commonly known as table salt [11].

Hypertension is one of the main causes of cardiovascular diseases, and reducing salt intake lowers blood pressure, therefore also decreasing the risk of cardiovascular disease [12]. Furthermore, salt reduction has an additive effect, direct and independent of the effect on blood pressure in cardiovascular diseases, for example, in reducing the risk of stroke [3,12]. In studies with long-term follow-up, results showed that a 2.5 g/day reduction in salt is associated with a 20% reduction in cardiovascular events [12].

The World Health Organization (WHO) recommends reducing salt intake to <5 g/day, although the average global adult intake is twice the recommended limit, about 10.06 g/day of salt [13]. The exact minimum daily requirement for salt is unknown but is estimated to be around 1.25–2.5 g. A salt intake of 5 g per day is sufficient to meet sodium and chloride needs in adults, as well as to reduce the risk of high blood pressure and heart disease [14].

A recent review identified bread, cereal and grain products and meat and dairy products as major contributors to dietary salt intake in most populations. It found that there is a wide variation in the consumption of discretionary salt across the world, depending on the economic development of the country. The contribution of discretionary salt to the diet ranged from less than 25% to more than 50% [15]. In the national food and physical activity survey carried out in Portugal, the main sources of salt were added salt (29.2%), the group of cereals, derivatives and tubers (20.6%) and the group of meat, fish and eggs (18.2%) [16].

The WHO has adopted the global target of a 30% reduction in average salt intake by 2025. About 75 countries have a national salt reduction strategy; most programs are multifaceted and include consumer education, target setting for salt content in foods, product reformulation, front-of-pack labeling schemes, interventions in public institutions and taxation of high-salt foods [17]. Interventions to reduce salt consumption must be tailored to individuals and subpopulations, as consumers are different in each country and the way in which salt is consumed is also different [18].

Better knowledge, attitudes and behaviors related to diet were associated with higher health status [19]. Therefore, its comprehension in relation to salt enhances the effect of interventions in changing behavior towards healthier choices [20].

The iMCSalt randomized controlled trial evaluated the impact of an intervention to reduce salt intake, which used a device to measure salt for cooking [21,22]. There was a decrease in sodium intake after the intervention, but without statistical significance, and a reduction in sodium and an improvement in the sodium/potassium ratio in hypertensive men in the intervention group, with statistical significance [22]. The aim of this study is to verify whether this intervention changed knowledge, attitudes and behavior towards salt and the main sources of salt.

## 2. Materials and Methods

### 2.1. Study Design and Participants

A detailed description of the study methods was previously published [21,22]. The study was registered at clinictrials.gov (NCT03974477).

The study was an 8-week randomized controlled trial with adults recruited from routine occupational health consultations at the University of Porto, which is located in the north of Portugal. Recruitment started in June 2019 and ended in January 2021 and was carried out by the doctor responsible for recruitment. Participants were assessed at baseline (week 0) and twice during the intervention (week 4 and week 8).

The following inclusion criteria were adult (>18 years old), report of motivation to control food consumption of salt and frequent home-cooked meals (more than 4 days a week and at least 3 Sundays a month). Exclusion criteria were hypotension, pregnancy, urinary incontinence, kidney disease, active infection that impacts kidney function, acute coronary syndrome, severe liver disease or heart failure, does not use salt to cook, and worker at the faculty promoting the study.

Randomization was performed after recruitment by a researcher independent of the recruitment process and intervention, and the allocation sequence was concealed until the initial assessment. Included participants were randomly allocated to the control or intervention group (ratio 1:1), using computerized random numbers and were stratified according to sex (ratio 1:1) and diagnosis of hypertension (ratio 0.4:0.6).

The intervention was the use of the Salt Control H equipment (Salt Control H—INPI, N° 20191000033265, prototype printed by fast printing) by participants at home to control and dose the amount of salt used in cooking all meals during the intervention period, and the researcher also provided some cooking strategies to prepare meals with adequate salt content. All participants, whether in the control group or in the intervention group, received the Portuguese food guide [23]. This guide consists of seven food groups with serving recommendations for each group.

All participants at the end of the study received information only about their nutritional status and anthropometric assessment.

### 2.2. Salt-Related Knowledge, Attitudes and Behavior

Participants were assessed according to the WHO STEPwise approach to surveillance of chronic disease risk factors [24].

The questionnaire consists of 13 questions. Three questions assess the behavior related to added salt: use of salt in meal preparation, discretionary use of table salt, and consumption of processed foods with high salt content, using a 5-point Likert scale (“always”, “often”, “often”, “sometimes”, “rarely”, “never”). Seven questions evaluated which behaviors were adopted to control salt intake with binary responses (“yes”, “no”), namely, avoid the consumption of processed foods, observe salt labels on food, eat meals without salt at the table, buy low-salt alternatives, cook meals without adding salt, use spices in addition to salt when cooking, and avoid eating out. Three questions related to knowledge and attitudes, including knowledge of the relationship between salt and health problems (“yes”, “no”), about the perception of salt consumption (“far too much”, “too much”, “just the right amount”, “too little”, “far too little”) and the importance of reducing salt intake (“very important”, “somewhat important”, “not at all important”).

### 2.3. 24-h Urinary Sodium Excretion

The 24-h urinary excretion collection was used as a proxy for salt intake.

Participants collected one urine sample at baseline and two urine samples during the intervention period (week 4 and week 8). Urine collection was performed on any day of the week, but preferably on Sunday. Participants received written instructions on the urine collection procedure and a coded sterilized container. On the day of collection, they rejected the first void and included all urine for that day and the first void for the following day, at the same time as that for the urine rejected the day before.

In urine, the following parameters were evaluated: sodium (by indirect potentiometry), creatinine (by photometry) and volume. The validity of the 24-h urine collection was analyzed through the relationship between urinary creatinine (mg/day) and body weight (kg). When creatinine (mg/day/kg) was <10.8 and >25.2 in women and <14.4 and >33.6 in men, samples were excluded [25].

The conversion of sodium from mmol to mg was performed by multiplying sodium (mmol) by 23 (mmol = mg/atomic weight). The conversion of sodium (Na) to salt (NaCl) was done by multiplying the sodium value by 2.542 (NaCl (g) = Na (g) × 2.542).

### 2.4. 24-h Dietary Recall

For the analysis of food groups, only those who ate cooked meals at lunch and dinner were considered, since the intervention focused on the reduction of added salt.

A 24-h dietary recall was used to estimate energy intake, sodium intake and major sources of sodium, including added salt. The analyzed day corresponded to the day of the 24-h urine collection. The questionnaire was administered by trained investigators and all food and beverage, commercial brands and cooking methods were questioned in detail and portion size estimated with the aid of a picture book. The conversion of food intake into nutrients was performed using the Food Processor Plus software (ESHA Research, Inc., Salem, OR, USA) adapted for the Portuguese population.

The foods were distributed into 12 food groups, adapted from the methodology of the national food and physical activity survey [16]: (1) cereals, cereal products and starchy tubers (paste, rice and other grains, potatoes and other starchy tubers, bread and rusks, flour, bread dough and pastry dough, infant cereals, breakfast cereals and cereal bars); (2) meat, fish and eggs (white meat, red meat, offal, processed meat, fish, crustaceans, mollusks and derivatives, processed fish and seafood); (3) dairy (milk, milk cream, yoghurt and other fermented milk, cheese); (4) soups (vegetable, meat, fish and chicken soups); (5) sweets, cakes and biscuits (table sugar, honey, molasses, syrup, jellies, jams, candied fruits, candy, gums, chewing gum, chocolates, chocolate snacks, ice cream, sweet desserts, cakes, cookies and biscuits); (6) salty snacks and pizzas (bread snacks, potato chips, salted popcorn, packaged fried snacks, patties, croquettes, codfish cakes, pies, puffed pastries and pizzas); (7) fruit, vegetables and vegetables (vegetables, nuts and oilseeds, fresh fruit and processed fruit); (8) non-alcoholic beverages (water, tea and infusions, coffee, natural and 100% fruit juices nectars, soft drinks, other non-alcoholic beverages); (9) other foods (yeasts, gelatins, flavors, herbs, spices, condiments, sauces, mayonnaise, powdered soups); (10) fats and oils (vegetable oils, olive oil, butter, margarines and minarines, other fats); (11) alcoholic beverages (wine, liquors, beer, spirits, other alcoholic beverages); (12) added salt.

Sodium in the foods of each food recall was divided by the corresponding food groups and its contribution was calculated in % terms, in relation to the sodium assessed by the 24-h urinary excretion. Cooked meals were introduced raw, according to the food yield by the cooking method. Added sodium was calculated by the difference between the sum of sodium values of foods in each group and the urinary sodium excretion value. After the calculations the sodium was converted to salt. Added salt is the salt added in the preparation and cooking, excluding the intrinsic sodium of raw foods.

### 2.5. Other Measures

Sociodemographic data were collected using a sociodemographic questionnaire based on the WHO STEPS questionnaire [24], physical activity level was assessed using the International Physical Activity Questionnaire-Short Form, validated and adapted for the Portuguese population [26], hypertension was diagnosed by the doctor responsible for the recruitment, and body mass index was calculated by Tanita MC180MA (Tanita, IL, USA), after entering the height of the participants measured by a stadiometer (portable stadiometer SEA 213, Hamburg, Germany) according to international standard procedures [27]. Physical activity was divided into moderate, vigorous and walking according to type, required energy expenditure (MET) and time spent (min). Physical activity level was considered high if total MET × min × week ≥3000, moderate ≥600 and low <600 [28].

### 2.6. Statistical Methods

The Kolmogorov-Smirnov test was used to test for normality. The statistics were performed according to the characteristics of the variables, the differences between the continuous independent variables were analyzed by the independent *t*-test (variables with normal distribution) or by the Mann-Whitney U test (variables with non-normal distribution) and for variables categorical the χ^2^ test. Differences between continuous dependent variables were analyzed using the Wilcoxon test and categorical variables were analyzed using the McNemar test. A Spearman’s correlation (non-normal distribution) was performed between urinary sodium excretion and sodium estimated by the food survey and between the sodium sources variables and the variables of the questionnaire on knowledge, attitudes and behavior in relation to salt. 

Statistical analysis was performed using the Statistical Package for the Social Sciences (SPSS Version 27). 

## 3. Results

### 3.1. Baseline Data

The study included 114 participants, of which 17 were excluded because they did not have valid urine at baseline and/or in the intervention period. A detailed description of the flow diagram of participants in the intervention to reduce salt intake has been published previously [22].

Table 1 shows the baseline characteristics of the participants. The mean age was 48, 52.6% were women and 40.2% were hypertensive. Most participants had higher education (86.6%), were married (68%) and has a moderate practice of physical activity (47.4%). On average, participants had a BMI of 25.9 kg/m^2^ and an energy intake of 2072 kcal/day. There were no significant differences between the intervention group and the control group.

### 3.2. 24-h Urinary Excretion and 24-h Dietary Recall

There were no significant differences between sodium calculated by 24 h urinary excretion at baseline (Me = 3025 mg) and sodium estimated by 24 h dietary recall (Me = 2670 mg), *p* = 0.204. At baseline, 45 participants were found to report sodium intake lower than that assessed by 24-h urinary excretion. The correlation between sodium estimated by 24 h urinary excretion and sodium estimated by 24 h dietary recall at baseline was statistically significant (r = 0.330, *p* = 0.003), Table 2.

In the assessments carried out during the intervention, no significant differences were found between the sodium calculated through the 24 h urinary excretion (Me = 3007 mg) and the sodium estimated by 24 h dietary recall (Me = 3318 mg), *p* = 0.514. In the assessments carried out during the intervention, it was found that 38 participants reported sodium intake lower than that assessed by 24-h urinary excretion. The correlation between the sodium estimated by 24-h urinary excretion and the sodium estimated by 24 h dietary recall in the assessments carried out during the intervention was not statistically significant (r = 0.190, *p* = 0.092), Table 2.

### 3.3. Sources of Salt Intake

In the analysis of the main sources of salt intake, 17 participants were excluded for not consuming cooked meals at lunch and dinner.

**Figure 1:**
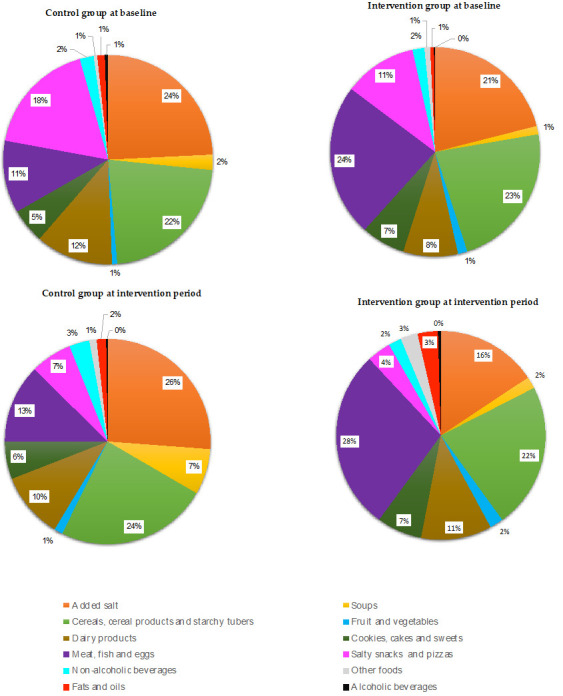
Food groups sources of salt in in the control and intervention group at baseline and in the intervention period.

At baseline, the main sources of salt in the control group were added salt (24%, $\bar{x}$ 3.3 g/salt), cereals, cereal products and starchy tubers group (22%, $\bar{x}$ 1.5 g/salt) and salty snacks and pizzas group (18%, $\bar{x}$ 0.9 g/salt), Figure 1, while in the intervention group, the main sources of salt were the meat, fish and eggs group (24%, $\bar{x}$ 2.1 g/salt), the cereals, cereal products and starchy tubers group (23%, $\bar{x}$ 1.6 g/salt) and added salt (21%, $\bar{x}$ 2.7 g/salt), Figure 1. There were significant differences between the control and intervention groups at baseline in the meat, fish and eggs group (*p* = 0.009).

At intervention period, the main sources of salt in the control group were added salt (26%, $\bar{x}$ 2.8 g/salt), the cereals, cereal products and starchy tubers group (24%, $\bar{x}$ 1.7 g/salt) and the salty snacks and pizzas group (13%, $\bar{x}$ 0.6 g/salt), Figure 1. In the intervention group, the main sources of salt were the meat, fish and eggs group (28%, $\bar{x}$ 2.1 g/salt), the cereals, cereal products and starchy tubers group (22%, $\bar{x}$ 1.4 g/salt) and added salt (16%, $\bar{x}$ 1.9 g/salt), Figure 1. There were significant differences between the control and intervention groups at intervention period in the salty snacks and pizzas group (*p* = 0.016). There were no significant differences before and after the intervention in any of the groups.

### 3.4. Salt-Related Knowledge, Attitudes and Behavior

After the intervention, participants in the intervention group reported a decrease in the addition of salt when cooking (*p* = 0.037). There was an increase in subjects in the intervention group who avoided the consumption of processed foods (from 54.2% to 83.3%, *p* = 0.001), who looked for salt on food labels (from 18.8% to 39.6%, *p* = 0.013), and who bought low-salt food alternatives (from 43.8% to 60.4%, *p* = 0.039). In the control group, the percentage of participants looking for salt on food labels increased (from 24.5% to 42.9%, *p* = 0.035), who purchased low-salt food alternatives (from 44.9% to 63.3%, *p* = 0.031), and who avoided eating out (from 49.0% to 65.3%, *p* = 0.039), Table 3. There were no significant differences between the intervention group and the control group at baseline and post-intervention assessments.

There were no significant differences between KAB in relation to salt and salt sources.

## 4. Discussion

As far as we know, this is the first study that assesses KAB towards salt during an intervention to reduce added salt during the cooking process.

The behavior related to salt after an intervention with a piece of equipment that measures the amount of salt for cooking improved in the intervention group, where there was an increase in the percentage of people who avoid eating processed foods, look for salt on food labels and buy food alternatives with less salt content. It was also found that participants in the intervention group reported adding less salt when cooking. There was no difference in knowledge about the health effects of salt and the importance of lowering salt intake, perhaps because participants already had a high level of knowledge.

The intervention did not include informative sessions that could increase the participants’ KAB about salt and the researchers never reported to the participants their level of salt consumption throughout the study, but only at the end, with the aim of not biasing the results. Therefore, every change in KAB related to salt resulting from this intervention must be carefully analyzed. 

Participants in the intervention group who reported they added less salt when cooking is in agreement with the aim of the present intervention study, which was to measure the amount of salt for cooking. This result is also in agreement with the analysis of the main sources of salt, where the salt added for cooking decreased in the intervention group.

In the control group, there was also an improvement in the behavior of controlling salt, there was an increase in the percentage of participants who look for salt on food labels, buy food alternatives with lower salt content and avoid eating out. The KAB questionnaire applied at baseline may have influenced the participants’ salt behavior, and that they knew they were involved in a salt study may have contributed to positive behavior change.

In other studies, after an intervention to reduce salt consumption, improvements were also observed. A multifaceted community-based salt reduction program using a Behavioral Impact Communication framework, lasting one year, in addition to an increase in the percentage of participants who limited their consumption of processed foods and looked at salt on food labels, also improved other behaviors such as limiting eating out, and using herbs and spices instead of salt [29]. In another 22-month consumer awareness campaign study they also significantly increased the percentage of participants who started looking at food labels, used herbs instead of salt and avoided eating out, but increased salt added during cooking [30]. In a year-long mass media campaign that focused on reducing discretionary salt consumption, there was an increase in the percentage of participants who reported that they reduced the addition of salt when cooking. Also, more participants reported that they stopped using salt, used more herbs, reduced their use of table salt, and more participants reported that reducing salt consumption is important [31].

These interventions were more intensive and focused on transmitting information and knowledge with different strategies and of longer duration (12 to 22 months) than the intervention in the present study. Yet the latter had positive results in changing the behavior of the participants in relation to salt, in addition to having increased the number of participants with the perception that they consumed too much or far too much salt. Previous studies [32,33] indicate that there is a tendency to have a positive perception about the quality of one’s diet, but with this intervention the participants increased their awareness of their salt consumption, which allows them to make more conscious choices. It is important to highlight that, although without statistical significance, the reported consumption of salt added to table food and industrialized foods rich in salt decreased after the intervention. There was also an increase in participants at the end of the intervention who stopped eating processed foods, ate foods without added table salt, used spices in addition to salt, avoided eating out, and recognized the importance of reducing salt in the diet.

A limitation of KAB questionnaires in relation to salt is the potential difference between reality and what is self-reported [34]. For example, some studies showed that when comparing self-reported behavior and what was observed, they concluded that people overestimated the use of nutrition labels [34,35]. Thus, in addition to the questionnaire, it is important to use biomarkers, such as urinary excretion. Biomarkers can be used to assess the difference between self-reported food intake and what is real, due to the bias in self-reported measures [36].

Added salt at baseline ranged from 21% to 24% in the intervention and control groups, respectively, a value below that mentioned in other articles in Portugal [16]. This reason may be related to the study sample having a high level of education. In a study of the change in salt intake of young adults over a mean follow-up time of 4.56 years, the reduction in salt intake was more pronounced in black adults of low and middle socioeconomic status, compared with a high socioeconomic status [37]. Added salt decreased in the intervention group after the intervention, but this was not significant. However, it is important to take into account that the average salt added at baseline was 2.7 g/day, the equipment provided 0.8 g per adult per meal, so in total the equipment dispensed 1.6 g/day for each adult. During the intervention period, the average intake of added salt decreased to 1.9 g/day, a value very close to the objective provided by the equipment, while the added salt in the control group increased. The snacks and pizzas group also showed a drop (7%), which coincides with the decrease in processed foods that participants reported. The meat, fish and eggs group increased by 4%, perhaps because more participants ate out less often. None of these changes were significant. In addition to added salt, the cereals and tubers group and the meat, fish and eggs group were the biggest contributors to salt intake, which is corroborated by other studies [15]. We found no relationship between salt sources and salt knowledge and behavior. Land et al. [38] also found no association between salt knowledge and behavior and measured salt intake.

A strength of the study was that sodium intake was assessed through 24-h urinary excretion, and two urine samples were taken and measured during the intervention period. Assessing sodium through urinary excretion is the gold standard method, because approximately 90–95% of ingested sodium is excreted in the urine [39]. Another strong point is that the recommendations and best practices of clinical trials were followed.

Our study has some limitations. First, data collection was carried out during the period of the COVID-19 pandemic, so we were unable to reach the sample size previously calculated and, due to confinements, the intervention time was longer in some participants. Second, most participants were highly educated, and the results are not extrapolated to the general population. Third, the questionnaire applied at baseline and knowing that they were involved in a study had influenced the participants’ behavior towards salt.

## 5. Conclusions

An eight-week intervention with a piece of dosage equipment to measure the amount of salt added when cooking improved salt behavior, but not knowledge and attitudes. After the intervention, in the intervention group more people reported not consuming processed foods, looking for salt on food labels, buying food alternatives with less salt and adding less salt when cooking.

The main sources of salt were added salt, cereals, cereal-based products and starchy tubers, the meat, fish and eggs group and the snack foods and pizzas group. After the intervention, it was found that the added salt decreased, but without statistical significance.

Evaluation of the level of knowledge, attitudes and behaviors at baseline and after an intervention is important to customize interventions and to understand what dimensions need to be improved.

## Figures and Tables

**Table 1:** Sociodemographic, lifestyle, clinical, anthropometric, and nutritional characteristics of 97 adults participating in an intervention to reduce added salt.

Participants’ Characteristics	Total (*n* = 97)	Intervention Group (*n* = 48)	Control Group (*n* = 49)	*p*-Value ^1^
Age (mean ± SD)	48 ± 10	46 ± 10	50 ± 10	0.065
Women *n* (%)	51 (52.6)	26 (54.2)	25 (51.0)	0.457
Education *n* (%)				0.393
No higher education	13 (13.4)	5 (10.4)	8 (16.3)	
Higher education	84 (86.6)	43 (89.6)	41 (83.7)	
Marital Status *n* (%)				
Single	17 (17.5)	10 (20.8)	7 (14.3)	0.281
Married	66 (68.0)	29 (60.4)	37 (75.5)	
Divorced	12 (12.4)	7 (14.6)	5 (10.2)	
Widow/er	2 (2.1)	2 (4.2)	0 (0.0)	
Hypertensive *n* (%)	39 (40.2)	20 (41.7)	19 (38.8)	0.772
Body mass index, kg/m^2^ (mean ± SD)	25.9 ± 3.9	25.5 ± 3.9	26.2 ± 3.9	0.384
Energy intake (kcal) (median [P25; P75])	2072 [1642; 2588]	2041 [1664; 2597]	2105 [1631; 2538]	0.931
Physical activity *n* (%)				
Low	35 (36.1)	16 (33.3)	19 (38.8)	0.846
Moderate	46 (47.4)	24 (50.0)	22 (44.9)	
High	16 (16.5)	8 (16.7)	8 (16.3)	

^1^ *p*-value calculated using the χ^2^ test on nominal variables, independent *t*-test on ordinal variables with normal distribution and Mann–Whitney U-test on ordinal variables with non-normal distribution. SD—Standard deviation; P25—25th percentile; P75—75th percentile.

**Table 2:** Descriptive data for sodium from 24-h urinary excretion and 24-h dietary recall.

Descriptive Data	Baseline		Intervention	
	Urinary Excretion (mg/day) *n* = 80	24 h Food Recall (mg/day) *n* = 80	Urinary Excretion (mg/day) *n* = 80	24 h Food Recall (mg/day) *n* = 80
Mean ± SD	3186 ± 1231	3124 ± 1688	3131 ± 1221	3318 ± 1724
Median	3025	2670	3007	3185
Minimum	1219	569	667	600
Maximum	5957	8938	6946	10,753
*p*-Value ^1^	0.204		0.514	

^1^ *p*-value calculated using the Wilcoxon test. SD—Standard deviation.

**Table 3:** KAB towards dietary salt in the intervention group and in the control group before and after an intervention to reduce salt intake.

Knowledge, Attitudes and Behaviors Regarding Salt Intake	Intervention Group			Control Group		
	Baseline	After Intervention	*p*-Value ^1^	Baseline	After Intervention	*p*-Value ^1^
Behavior related to added salt						
Add salt to food (often or always) (%)	2.1	0.0	0.589	2.0	0.0	0.558
Add salt while cooking (often or always) (%)	93.7	81.2	0.037	91.8	85.7	0.078
Consume processed foods high in salt (often or always) (%)	37.5	31.3	0.221	38.7	24.5	0.119
Knowledge and attitudes						
Perceived salt consumption (too much or far too much) (%)	14.6	27.1	0.142	16.3	12.2	0.432
Importance of lowering salt in the diet (Not at all important) (%)	10.4	4.2	0.617	6.1	4.1	0.405
Agreed that too much salt could cause health problems (no) (%)	2.1	10.4	0.250	2.0	6.1	0.625
Behaviors to reduce salt						
Avoid consuming processed foods (yes) (%)	54.2	83.3	0.001	67.3	77.6	0.070
Look at the salt labels on food (yes) (%)	18.8	39.6	0.013	24.5	42.9	0.035
Eat meals without adding salt at the table (yes) (%)	87.5	91.7	0.687	95.9	89.8	0.687
Buy low-salt alternatives (yes) (%)	43.8	60.4	0.039	44.9	63.3	0.031
Cook meals without adding salt (yes) (%)	29.2	29.2	1.000	20.4	26.5	0.508
Use spices other than salt when cooking (yes) (%)	62.5	75.0	0.180	65.3	63.3	1.000
Avoid eating out (yes) (%)	35.4	54.2	0.064	49.0	65.3	0.039

^1^ *p*-value calculated using McNemar test on nominal variables and the Wilcoxon test on ordinal variables. All answer options in Supplementary Materials, in Tables S1 and S2.

## Data Availability

The data presented in this study are available on request from the corresponding author.

## Supplementary Materials

The following supporting information can be downloaded at: https://www.mdpi.com/article/10.3390/foods11070981/s1, Tables S1 and S2: Knowledge, attitudes and behavior dietary salt in the intervention group and in the control group before and after an intervention to reduce salt intake.
