# Supplementary material for: Salt-Related Knowledge, Attitudes and Behavior in an Intervention to Reduce Added Salt When Cooking in a Sample of Adults in Portugal
Source: Foods. 2022 Mar 28;11(7):981. doi: 10.3390/foods11070981 (PMC8997494; doi:10.3390/foods11070981)
Supplement: Supplementary file 1 [file foods-11-00981-s001.zip › foods-1602231-supplementary.pdf]

Table S1. Knowledge and behavior dietary salt in the intervention group and in the control group before and after an intervention to reduce salt intake.

Knowledge, attitudes and behavior towards dietary salt		Baseline		Intervention period	
		Intervention n (%)	Control n (%)	Intervention n (%)	Control n (%)
How often do you add salt to your food before you eat it or as you are eating it?	Always	1 (2.1)	1 (2.0)	-	-
	Often	-	-	-	-
	Sometimes	2 (4.2)	-	1 (2.1)	1 (2.0)
	Rarely	3 (6.3)	8 (16.3)	6 (12.5)	7 (14.3)
	Never	42 (87.5)	40 (81.6)	41 (85.4)	40 (81.6)
p		0.494		0.785	
How often is salt added in cooking or preparing foods in your household?	Always	34 (70.8)	39 (79.6)	29 (60.4)	28 (57.1)
	Often	11 (22.9)	6 (12.2)	10 (20.8)	14 (28.6)
	Sometimes	3 (6.3)	3 (6.1)	5 (10.4)	6 (12.2)
	Rarely	-	1 (2.0)	2 (4.2)	-
	Never	-	-	2 (4.2)	-
p		0.397		0.861	
How often do you eat processed food high in salt	Always	1 (2.1)	6 (12.2)	1 (2.1)	2 (4.1)
	Often	17 (35.4)	13 (26.5)	14 (29.2)	10 (20.4)
	Sometimes	23 (47.9)	22 (44.9)	20 (41.7)	27 (55.1)
	Rarely	7 (14.6)	8 (16.3)	12 (25.0)	9 (18.4)
	Never	-	-	-	-
	Don’t know	-	-	1 (2.1)	-
p		0.730		0.831	
How much salt do you think you consume?	Far too much	1 (2.1)	1 (2.0)	-	-
	Too much	6 (12.5)	7 (14.3)	13 (27.1)	6 (12.2)
	Just the right amount	18 (37.5)	26 (53.1)	24 (50.0)	27 (55.1)
	Too little	12 (25.0)	10 (20.4)	9 (18.8)	7 (14.3)
	Far too little	2 (4.2)	4 (8.2)	2 (4.2)	2 (4.1)
	Don't know	9 (18.8)	1 (2.0)	-	6 (12.2)
p		0.084		0.070	
Do you think that too much salt in your diet could cause a serious health problem?	Yes	46 (95.8)	47 (95.9)	40 (83.3)	44 (89.8)
	No	1 (2.1)	1 (2.0)	5 (10.4)	3 (6.1)
	Don't know	1 (2.1)	1 (2.0)	3 (6.3)	2 (2.0)
p					
How important to you is lowering the salt in your diet?	Very important	29 (60.4)	32 (65.3)	30 (62.5)	27 (55.1)
	Somewhat important	14 (29.2)	14 (28.6)	16 (33.3)	19 (38.8)
	Not at all important	5 (10.4)	3 (6.1)	2 (4.2)	2 (4.1)
	Don´t know	-	-	-	-
p		0.504		0.557	

Table S2. Knowledge and behavior towards dietary salt in the intervention group and in the control group before and after an intervention to reduce salt intake.

Knowledge, attitudes and behavior towards dietary salt		Baseline		Intervention period	
		Intervention n (%)	Control n (%)	Intervention n (%)	Control n (%)
Avoid/minimize consumption of processed foods	No	22 (45.8)	16 (32.7)	8 (16.7)	10 (20.4)
	Yes	26 (54.2)	33 (67.3)	40 (83.3)	38 (77.6)
p		0.184		0.601	
Look at the salt or sodium labels on food	No	39 (81.3)	37 (75.5)	29 (60.4)	27 (55.1)
	Yes	9 (18.8)	12 (24.5)	19 (39.6)	21 (42.9)
p		0.493		0.679	
Eat meals without adding salt at the table	No	6 (12.5)	2 (4.1)	4 (8.3)	4 (8.2)
	Yes	42 (87.5)	47 (95.9)	44 (91.7)	44 (89.8)
p		0.132		0.643	
Buy low salt/sodium alternatives	No	27 (56.3)	27 (55.1)	19 (39.6)	17 (34.7)
	Yes	21 (43.8)	22 (44.9)	29 (60.4)	31 (63.3)
p		0.909		0.673	
Cook meals without adding salt	No	34 (70.8)	39 (79.6)	34 (70.8)	35 (71.4)
	Yes	14 (29.2)	10 (20.4)	14 (29.2)	13 (26.5)
p		0.318		0.820	
Use spices other than salt when cooking	No	18 (37.5)	17 (34.7)	12 (25.0)	17 (34.7)
	Yes	30 (62.5)	32 (65.3)	36 (75.0)	31 (63.3)
p		0.774		0.266	
Avoid eating out	No	31 (64.6)	25 (51.0)	22 (45.8)	16 (32.7)
	Yes	17 (35.4)	24 (49.0)	26 (54.2)	32 (65.3)
p		0.176		0.210	
